# An Atypical Case of Transverse Diverticulitis and the Changing Management of Diverticular Disease

**DOI:** 10.51894/001c.6979

**Published:** 2018-09-26

**Authors:** Andrew C. Ostosh, Adeeb Saleh, Kevin M. Boehm

**Affiliations:** 1 College of Human Medicine, East Lansing, MI; Department of Emergency Medicine PGY1 Resident, Saginaw, MI Michigan State University; Central Michigan University; 2 Department of Emergency Medicine Chief Resident, Livonia MI; College of Osteopathic Medicine, East Lansing, MI St Mary Mercy Livonia; Michigan State University; 3 College of Osteopathic Medicine, East Lansing MI; Department of Emergency Medicine, Director of Education, Fort Lauderdale, FL Michigan State University; Broward Health Medical Center

**Keywords:** non-surgical management of diverticulitis, antibiotic management, transverse diverticulitis, diverticulitis

## Abstract

Diverticulitis is an inflammation of an out pouching of the lower gastrointestinal tract, particularly in the large intestine. Although the condition is taught to medical students as typically occurring in the left lower quadrant of the abdomen, right-sided and transverse forms diverticulitis can occur. Uncomplicated, e.g., non-perforated, diverticulitis is usually treated with antibiotics. Complicated, e.g. perforated, is usually treated with surgery. The purpose of this case report is to present an atypical case of perforated diverticulitis and review current recommendations for this condition. This was a case of transverse diverticulitis in a man in his late 40’s who recovered with non-operative treatment. The widespread use of computerized tomography (CT) scans makes diagnosing diverticular disease relatively simple, but treatment is evolving. The case summarized here shows that less invasive measures can be used in treating both complicated and uncomplicated diverticular disease. After an uncomplicated in-patient admission for intravenous antibiotics, the patient was discharged in stable condition with a prescription for oral antibiotics and clinic follow-up. Classic medical school teaching concerning treatments for complicated and uncomplicated forms of diverticulitis have been updated but require further research testing.

## INTRODUCTION

Diverticulum are the out-pouching of the large intestine. When these out pouches become inflamed, the resulting painful condition is usually located in the lower left quadrant of the abdomen. The number of hospital admissions due to diverticular disease is increasing in industrialized nations, with an increase of 26% reported in a relatively recent seven-year period.^1^ Typically, diverticulitis presents with clinical signs and symptoms that make it relatively easy to diagnose: left lower quadrant abdominal pain, nausea, vomiting, and anorexia. However, atypical presentations do exist. For example, right sided diverticulitis is more commonly seen in Asian populations.^2^ In very rare cases, the disease has also been reported in the transverse colon.^3–5^

## METHODS

### Case Report

A man in his later 40's with a history of type 2 diabetes presented to a community-based emergency department in Michigan complaining of moderate epigastric pain. The pain, which he described as constant, cramping and pressure-like with no radiation, had been gradually increasing over the past three days. Movement did not affect his pain. While he did not associate his pain with eating, he reported a decreased appetite over the same time frame. He had not tried any medications to manage his pain.

The patient had been diagnosed with type 2 diabetes one year prior and stated that his blood sugars were well controlled with his insulin doses. He had also been diagnosed with diverticulitis 10 years prior which was treated with antibiotics. After treatment, a colonoscopy was performed, which was unremarkable according to the patient. He denied any surgical history. Previously that same day, the patient had spent eight hours in a car traveling from a wedding. He reported increased alcohol consumption at the wedding the night prior. The patient stated that he typically consumes seven drinks a week and was a nonsmoker. He denied any other drug use. He often lifted heavy items for work as a beer distributer but reported no recent work-related injuries.

The patient’s vital signs on arrival were a heart rate of 119 beats per minute, a blood pressure of 163/100 mmHg., a respiratory rate of 16 and a temperature of 36.9 C. During the physical examination, the patient was found to be restless on the hospital bed, with clear lung sounds and tachycardia, but regular heart sounds. An abdominal exam found decreased bowel sounds, mild tenderness to deep palpation in the epigastric and left upper quadrant regions, and positive rebound tenderness in the left lower quadrant region. The remainder of the physical exam was unremarkable. Patient was given a 1 liter Lactated Ringer bolus and 2 mg morphine intravenous (IV) for pain.

**Figure 1:**
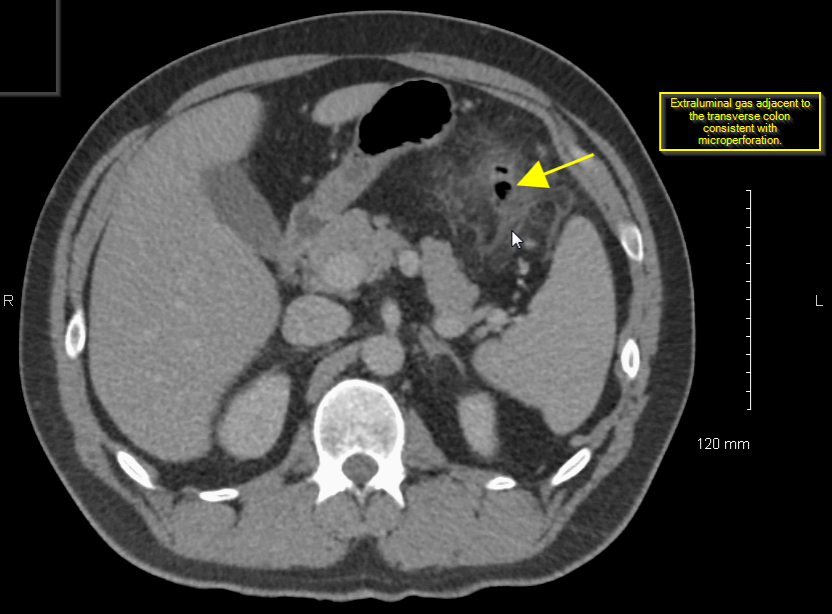
CT with Contrast of Patient Showing Transverse Diverticular Disease with Micro-Perforation (Yellow Arrows) Obtained in ED

Laboratory work was significant for leukocytosis with a 12.7 thou/mcl white blood cell count, absolute neutrophils of 9.7 thou/mcl and a lactic acid of 2.2 mMol/L. Table 1 has a complete list of lab values with abnormal labs appearing in bold font. Due to the patient’s lactic acidosis, persistent tachycardia, and left lower quadrant rebound tenderness, a computerized tomography (CT) scan of the abdomen was obtained to look for any acute abnormalities with strong suspicion of complicated sigmoid diverticulitis. Because of the authors’ high suspicion that the patient had an infectious process, he was given 500 mg of IV Metronidazole and 400 mg of IV Ciprofloxacin prior to the CT scan. The results of the CT scan (Figure 1) demonstrated acute diverticulitis located along the distal aspect of the transverse colon with signs of micro-perforation.

**Table 1:** A List of Laboratory Values on Admission to the Emergency Department

**Lab**	**Patient**	**Reference value**
**Sodium**	**135 mMol/L**	136-145
**Potassium**	4.8 mMol/L	3.5-5.3
**Chloride**	103 mMol/L	98-107
**Carbon Dioxide**	23 mMol/L	21-32
**Glucose**	129 mg/dL	60-100
**BUN**	12.2 mg/dL	8.0-22.0
**Creatinine**	1.08 mg/dL	0.60-1.20
**Calcium**	9.0 mg/dL	8.5-10.1
**Alkaline Phosphatase**	68 Units/L	46-116
**ALT**	28 Units/L	12-58
**AST**	**44 Units/L**	10-37
**Bilirubin Total**	**1.2 mg/dL**	0.1-1.0
**Bilirubin Direct**	0.1 mg/dL	0.0-0.3
**Albumin**	3.9 gm/dL	3.4-5.0
**Lipase**	92 Units/L	73-393
**Magnesium**	1.9 mg/dL	1.8-2.4
**Lactic Acid**	**2.2 mMol/L**	0.4-2.0
**Hemoglobin**	15.4 gm/dL	13.5-17.5
**Hematocrit**	48.6%	37.6-52.0
**WBC**	**12.7 thou/mcL**	3.6-11.1
**Neutrophil Absolute**	**9.7 thou/mcL**	1.7-7.6
**Lymphocyte Absolute**	1.9 thou/mcL	0.8-3.3
**Platelet**	282 thou/mcL	140-450

*Abnormal lab results appear in bold font

The General Surgery service was consulted. Since the patient appeared non-toxic, the surgeon opted to manage non-surgically with serial abdominal examinations, monitoring, and continuation of IV antibiotic therapy. The patient responded well to this conservative therapy, and was discharged three days later in stable and improved condition on oral amoxicillin/clavulanate 875mg/125mg twice daily for 14 days with follow up with the surgeon. The authors attempted to contact the patient to learn the results of the follow up appointment, but were unable to contact them.

## DISCUSSION

Diverticulitis can be classified as either complicated or uncomplicated. Uncomplicated diverticulitis is defined as localized inflammation in the mucosa, submucosa or peri-colonic fatty tissue without perforation. Complicated diverticulitis is defined as diverticulitis with formation of an abscess, perforation or fistula. Regardless of type, diverticulitis is typically a disease of the sigmoid colon. This is due to the increased pressures associated with the sigmoid colon.

As Laplace’s Law states, pressure is equal to tension divided by radius.^6^ The small radius of the sigmoid colon results in increased pressure that can contribute to diverticular disease development. However, this theorem does not exclude the presence of diverticulitis in other areas of the colon. The second most common presentation is the right lower quadrant with ascending colonic disease.^7^ This is often confused with appendicitis and much remains unknown about this disease presentation. The prevalence of diverticulitis in the lower right quadrant is also unknown, but it typically occurs in young males rather than elderly females, the population in which sigmoid diverticulitis typically presents.^7^

The presentation of this case was strongly suggestive of complicated diverticulitis, but with the physical exam findings, it was expected to be in the sigmoid colon, not the transverse colon. The ease and availability of the CT scan makes the diagnosis of diverticulitis easy for the clinician, but the same cannot be said for choosing a course of treatment. Management of diverticulitis is evolving, and research is being done to validate the use of more conservative measures in the treatment of both complicated and uncomplicated diverticulitis.^8–10^

The mainstay treatment of uncomplicated diverticulitis has been IV antibiotics. However, recent literature indicates that this may not be supported by current evidence-based medicine.^8^ The authors reviewed four sets of guidelines set by the Society for Surgery of the Alimentary Tract, the American Society of Colon and Rectal Surgeons, the European Association for Endoscopic Surgery and the American College of Gastroenterology. They determined that all guidelines recommend using antibiotics but only two, the American Society of Colon and Rectal Surgeons and the American College of Gastroenterology, referenced the original research.^3^

The results of newer studies have tested antibiotic treatment of uncomplicated diverticulitis versus supportive measures to gauge the true effectiveness of antibiotics.^9,10^ One of the more promising studies includes a randomized clinical trial which enrolled a total of 620 Islandic patients diagnosed with uncomplicated diverticulitis via CT scan. Splitting the patients into two groups, one group received IV then oral antibiotic therapy and the other received only supportive measures. The two groups showed no significant difference with regards to complications or need for surgical intervention. Three patients in each group (i.e., 1.9% in non-antibiotic group, 1.0% in antibiotic group) suffered from perforation while three patients in the supportive treatment group developed an abscess.^10^

The effective management of complicated forms of diverticulitis is further changing. The earlier gold standard of emergent surgical intervention is now evolving toward aggressive non-operative management.^11^ This is due to advancing medical technologies and an improved understanding of diverticulitis complications. Percutaneous abscess drainage by interventional radiology has been shown to lessen the complexity of diverticulitis.^2^ Also, colonoscopy is being further utilized for clip placement and cautery can reduce the need for surgery in hemorrhagic diverticulitis.^2^ Both of these interventions may act as a bridge to surgery, but in less severe cases, they may serve as solitary treatment when combined with antibiotics.^2^ A notable 2011 retrospective review identified 136 patients with perforated diverticulitis. In this sample, only five (3.7%) required emergent surgery and seven (5.1%) required urgent surgery after failing non-operative management. A total of 124 of the 136 patients were successfully treated non-operatively, resulting in a 91% effectiveness of non-operative treatment in this sample.^11^

## CONCLUSIONS

Although it is relatively easy to diagnose diverticulitis, deciding on the optimal course of treatment can be more complex. The treatment of this condition is changing rapidly. Recent studies have indicated that less invasive measures can be effective in treating the disease. More research needs to be completed, however, to further understand the true benefits of these less invasive measures.

Just as knowledge of how to best treat diverticular disease is evolving, more effective ways of stratifying diverticular disease are needed to standardize practice guidelines for clarifying the risks and benefits of evolving treatments. It may be time to expand from the classic complicated and uncomplicated nomenclature and look instead to a differential classification based on lab results, CT changes, and responses to management to better risk stratify future patients.

### Conflict of Interest

The authors declare no conflict of interest.
